# Factors Affecting Car Sickness of Passengers Traveled by Vehicles in North Shewa Zone, Oromia, Ethiopia

**DOI:** 10.1155/2022/6642603

**Published:** 2022-02-22

**Authors:** Zelalem Tadese, Berhanu Teshome, Endeshaw Mengistu

**Affiliations:** ^1^Department of Sociology, Salale University, P.O. Box 245, Fitche, Oromia, Ethiopia; ^2^Department of Statistics, Salale University, P.O. Box 245, Fitche, Oromia, Ethiopia

## Abstract

**Introduction:**

Car sickness is the state of being unhealthy as a result of motions that occur while traveling by vehicles. Passengers traveled by vehicles had experienced car sickness not only as a result of the biological effects but also other associated factors. Therefore, this study aimed to identify sociocultural, individual behavioral factors and situational factors resulting in car sickness of passengers traveling by minibus or bus or both.

**Methods:**

This study was designed in a cross-sectional study and employed a quantitative approach to collect data among 384 passengers. Primary data were collected by a survey method. Both adult male and female passengers without any confirmed disease participated in the study. Car sickness was measured as whether a passenger traveling by vehicle in the past six months had at least one of the signs and symptoms either vomiting, nausea, headache, and (cold) sweating. Quantitative data were analyzed using descriptive statistics, bivariate analysis, and binary logistic regression. The multivariate logistic regression model was employed and used to execute the associated risk factors by declaring all statistical tests significantly at *p*-value ≤0.05.

**Results:**

The results indicated that being older (aOR = 0.972, 95% CI: 0.947, 0.999) and male passengers (aOR = 0.357, 95% CI: 0.190, 0.673) significantly decreased occurrences of car sickness. However, sleep deprivation (aOR = 8.540, 95% CI: 2.575, 28.328), eating heavy meals before starting traveling (aOR = 4.147, 95% CI: 1.659, 10.366), the aggressiveness of drivers (aOR = 5.467, 95% CI: 2.456, 12.172), and travel with other passengers in overcrowded vehicles (aOR = 9.5212, 95% CI: 5.194, 17.455) were significantly contributed to car sickness.

**Conclusions:**

The findings suggested that younger passengers should take medications that reduce the sensation of car sickness before starting traveling and female passengers should reduce unpaid domestic work before their travels. In order to prevent or reduce the sensation of car sickness, passengers should take enough physical rest, sleep well, and avoid eating heavy meals before a journey. Furthermore, passengers should strongly advise or kindly request aggressive drivers to drive slowly and uniformly. Last, the passengers should avoid traveling with other passengers in overcrowded vehicles as much as possible.

## 1. Introduction

Motion sickness is a state of being unhealthy that passengers could experience when they travel by vehicles, boats, and aircrafts [1–3]. Car sickness is a type of motion sickness that ocurs in road vehicles [1, 4, 5]. Furthermore, car sickness is a hostile event that occurs when passengers travel certain distances in vehicles [6–8]. Motion sickness in general and car sickness, in particular, are manifested by assured signs and symptoms. In this fashion, in the early stages, car sickness is described by pallor, restlessness, and (cold) sweating [9, 10]. In later stages, car sickness is illustrated by salivation, dizziness, nausea, vomiting, and other physical discomfort [3, 9, 10].

Globally, 66.7% of travelers experienced car sickness at some point in their life [1, 5, 11]. Car sickness is a universal experience that passengers face during travels by vehicles [12, 13]. Generally, women are more susceptible to car sickness than men [1, 8, 14–16]. Such differences between them were observed as a result of the sociocultural differences like social roles and social norms toward the type of work performed, not the biological one^3^. Car sickness was more prevalent in younger people than in older people as a result of the physiological effects [1, 2, 17]. Explicitly, individuals with the age between 2 and 12 years had more car sickness than others [2].Conversely, individuals with the age of 50 years and above had rarely experienced car sickness [2]. Furthermore, passengers with and without health problems experienced car sickness owing exposure to the unusual situations [18–21]. Nevertheless, passengers who are sick or ill experience more car sickness than those without disease or illness [6, 22].

Great concerns about car sickness in vehicles were given by previous investigations [1, 3, 15, 23]. The main reasons for the concerns were that vehicles increasingly accounted for the transportation services of the vast majority of passengers. In addition, car sickness was negatively affecting passengers' comfort and well-being [3, 19]. The automated vehicles developed in reaction to the manual one with the aim at reducing car sickness, although they significantly increased the likelihood of car sickness [5, 9, 11, 23]. Furthermore, in automated vehicles, drivers are passengers, and they experience car sickness but not like the passengers [24].

Several existing studies investigated contributory factors to car sickness. For instance, it occurred as a result of a mismatch between actual and anticipated sensory signals, and a mismatch between acceleration and deceleration of motion by vehicles [3, 5, 14, 15]. From these studies, it was arguable that car sickness was associated with physiological effects of the body. Car sickness occurred due to the contributed factors such as gender, age, and seating positions at the back within the vehicles[11, 13, 17]. However, herein, limited sociodemographic variables like age and sex were identified as contributory factors to car sickness. In addition to gender and age variables, the personal and individual behavior of passengers was not considered for their contribution to car sickness. Furthermore, car sickness was associated with a health problem or disease [6, 22]. The current study tried to use the existing literature on car sickness in general in Ethiopia and in particular in the study area. Unfortunately, searching with all efforts and necessary methods, it was noticeable as there were no studies (till the date of writing this study) in Ethiopia.

The causality of a disease or an illness was much more complex than a single causal factor [25]. Along this line, it was strongly suggested that contemporary researchers should use the multicausality theory to investigate contributory factors that affect health and well-being of individuals [25]. Actually, the multicausal theory was first developed in 1973 by Dr. Mervyn Susser [26]. Susser developed the theory by stating that practices of health behaviors do not exist in a vacuum apart from individuals and the casualties of diseases or illnesses exist within ways of life and in societal patterns or structures . Briefly, the theory was emphasized on physiological, psychological, environmental, cultural, and social factors as they have the ability to shape and determine the patterns of well-being and health of individuals.

An identification of multicausality factors is an important step toward designing effective preventive measures. The existing studies treated the casualties of car sickness as if only from the physiological effect and underscored only limited sociodemographic variables [1, 3, 5, 6, 11, 14, 15, 17, 22]. However, the monocausal approach used in the existing studies resulted in a restricted capacity to identify the range of contributing factors to car sickness among the passengers. Besides, there is a limit of evidence or no detailed description of the multicausal approach of car sickness in the study area. Consequently, such gaps decrease the potential of individuals and policy makers to use the existing literature to design effective preventive measures using holistic and multilevel approaches. Hence, the current study framed a multicausality approach to investigate a wider understanding of contributory factors to car sickness.

Applying either preventive measures or interventions to reduce psychological and physical discomfort resulting from car sickness is more effective if all programs stress the multicausal approach by contextualizing evidence. To be cognizant of such types of gaps and limited evidence, this study identified the relative effects of sociocultural, situational, and individual behavioral factors on car sickness.

## 2. Materials and Methods

### 2.1. Study Setting, Design, and Period

The study was carried out in randomly selected five areas (Wachale, Debre Libanos, Fiche, Kuyu, and Warra Jarso) from thirteen woreda and one town administration in North Shewa Zone, which is one of the zones in Oromia Regional State. The zone is found in the North of Addis Ababa (the capital city of Ethiopia). At each district with different sizes, there is a bus station or terminal which is serving people for the transportation services. The study was conducted among passengers who are traveling by vehicles from one place to another place. A vehicle is a minibus or a bus that is served to take people from one place to another.

Since it was difficult to contact passengers who were waiting to travel by vehicles to another place in the centers of selected study areas, the researchers were forced to search for places where passengers were actually available. Then, researchers communicated with the heads of the transport offices of each selected study area through phone to find out where the passengers in the towns actually waited to travel by vehicles. The bus stations or travel hubs were suggested as study areas by all heads of the offices. At each study area, with different sizes there was a bus station or a travel hub which served the population for the transportation services. A community-based cross-sectional study design was employed to investigate the contributory factors associated with passengers' car sickness. The study was carried out between October 2019 and November 2019.

### 2.2. Inclusion and Exclusion Criteria

The study participants were both adult male and female passengers who were waiting to travel by vehicles in the bus station and with no history of disease during the study carried out in the study area. Young passengers who were under the age of 18 year and passengers who were medically on treatment during the study were not included in the study. Last, the drivers and assistant drivers were not included in the study.

### 2.3. Sample Size Determination and Sampling

A single population formula was used to determine the sample size. Accordingly, the formula for sample size determination used was *n* = [*p* (1 ‒ *p*)] *∗* [Z*α*/2)2/(e)2], where *n* denotes the sample size, Z*α*/2 is the reliability coefficient of standard error at the 5% level of significance, which is equal to 1.96, and *p* represents 50% proportion to experience car sickness among passengers since no previous study on this topic, with a 95% confidence coefficient [27]. Hence, the calculation yielded a sample size of 384 passengers that are computed as: *n* = [0.5 *∗* 0.5] *∗* [1.96 *∗* 1.96]/[0.05 *∗* 0.05] = [0.25 *∗* 3.8416]/0.0025 = 0.9604/0.0025 = 384.16. Then, the sample size was allocated to all sites in equal proportions. Based on the determined sample size, the samples computed were 77 for all sites except 76 at Debre-Libanos.

A multistage sampling technique was used. Using the primary sampling units, random sampling was used to select five study areas from the fourteen identified study areas because all of the study areas were assumed to be having similar attributes in using vehicles as a means of transportation services. In the secondary sampling units, passengers were selected randomly in each bus station or bus terminal. The sample size was proportionally allocated to each selected study area. Finally, a simple random sampling was employed until the allocated sample size reached.

### 2.4. Study Participants and Sample

The study participants were the passengers who waited for traveling in the bus station in the study sites. Thus, the samples were from different bus stations or terminals of each selected town.

### 2.5. Study Instrument

The study was conducted using an interview schedule instrument with close-ended type questions. The interview schedule, which took around 10 minutes, was carefully and logically prepared. The instrument involves four components of associated risk factor measurement: the first item consists of sociodemographic characteristics, the second item involves sociocultural factors, and the third item encompasses the nature of travel or journeys and followed by the individual behavioral factors. The questions were pretested among fifty nonsampled passengers in Fiche town where Salale University is located. Some language and formatting adjustments were made based on the pretest findings. Then, primary data were collected by the trained data collectors from all sites using an interview schedule instrument.

A common instrument used to assess multiple dimensions of motion sickness is a Motion Sickness Assessment Questionnaire (MSAQ) [7]. The tool inquiries associated factors to motion sickness that resulted from different types of means of transport (e.g. minibus, car, bus, airplane) and the tool employs to assess the previous occurrences of motion sickness and individuals' differences toward car sickness and/or travel-related feeling unwell. Further, the tool was validated for passengers using different vehicles for a means of transportation services irrespective of their geographical location or environment. Along these lines, this study developed respective questions which help to meet the objectives.

## 3. Procedure

This study separately assessed the incidence of car sickness through the assured signs and symptoms such as vomiting, nausea, headache, and cold sweating. This study assessed the occurrence of the signs and symptoms, while passengers traveled by vehicles using a forced choice, yes/no questions in the preceding six months. Study participants who answered yes were considered as they had car sickness; others who answered no were assigned to groups without car sickness. This study assessed the contributory factors to car sickness using a modified version of interview schedule, which included 21 items with different response patterns. The questions were translated into Afaan Oromo and Amharic, which are commonly spoken languages in the study areas. After completing the informed consent (verbal) procedure, study participants were informed briefly about the purposes of the study. Then, the study participants were asked questions, which are logically and carefully prepared for their responses. Following similar procedures, data were collected until reaching the needed samples.

### 3.1. Measurement of Variables

Car sickness was manifested by assured signs and symptoms like vomiting, nausea, headache, and (cold) sweating [4, 6, 8, 28]. In this study, car sickness was measured whether passengers traveled by vehicles in the past six months had one of the signs and symptoms mentioned above. Therefore, a passenger who reported at least one of the signs and symptoms during traveling is said to be experiencing car sickness. Thus, the outcome variable, car sickness, was categorized into binary responses as follows:

(1) $$\begin{array}{c} Y=\left\{\begin{array}{c} 0\text{ if passengers had not experienced one of the signs and symptoms over }6\text{ months,}\\ 1\text{ if passengers had experienced at least one of the signs and symptoms over }6\text{ months}. \end{array}\right. \end{array}$$

The passengers were asked as they had signs and symptoms in the past six months while traveling from one place to another place by vehicles. This study measured car sickness if the abovementioned four indicators fulfilled the following definitions. First, with nausea, the car sickness of study participants can be measured if they felt conditions that made them have a lousy taste and sense to vomit. Second, during travel, if study participants had reflexed the act of ejecting the contents that were eaten or drunk before traveling from their stomach through the mouth, it was inferred as vomiting. Third, after starting traveling, when the study participants had faced unhappiness, troubles, or worrying about a trip, they recognized that they developed a headache. If the study participants behaved in circumstances that resulted in discomfort and contributed to the irritation while traveling, they experienced car sickness. Finally, under the normal state of conditions, if the study participants perspired on their face or body because they were frightened about the situation of journeys, they had experienced car sickness. The details of indicators of car sickness are presented in Table 1. In this study, the independent variables can be broadly categorized into sociocultural, individual, behavioral factors, and nature of travels. The details of the factors are presented in Table 2.

### 3.2. Statistical Analysis

The distribution of sampled passengers was presented and explained by descriptive statistics such as frequencies and percentages. To estimate the effects of the sociocultural factors, nature of journeys that were considered as situational factors, and individual behavioral factors associated with car sickness, this study employed a binary logistic regression analysis with the odds ratio with a 95% confidence coefficient. The data were analyzed using SPSS software version 20.

The Pearson chi-square test was employed in the bivariate analysis to test the association between predictors and car sickness. Then, all significant predictors with a *p*-value <0.25 in the bivariate analysis were included in the multivariate logistic regression analysis. For all statistical tests, the alpha levelwas set to 0.05.

### 3.3. Ethics Approval and Considerations

The study got an official permission letter numbered SLU/632/19/2012 from the Office of Salale University Vice President for Research and Community Services for the investigations and inquiries. Further, verbal consent was sought from each study participant.

## 4. Results

### 4.1. Sociodemographic Characteristics of Study Participants

Study participants who had at least one of the assured signs and symptoms in the past six months were assigned to groups who had experienced car sickness. All others were assigned to groups without experiencing car sickness.  Table 3 indicates that 129 (33.6%) of the study participants were females, while 255 (66.4%) of them were males. In line with age categories, the study participants less than the age of 20 years were 42 (10.9% ) and from the age of 20 to 40 years were 172 (44.8%), while 98 (25.5%) of them were from the age of 41 to 60 years. Last, 72 (18.8%) of the study participants were above the age of 60 years. Related to the occupations, 103 (26.8%) of the study participants were merchants, while 135 (35.2%) of them were governmental employees. Apart from this, 89 (23.2%) of the study participants were passengers who sought work, and 57 (14.8%) of them were students who attended college and above.

### 4.2. Indicators of Car Sickness

Considering the history that went back to six months, this study assessed whether passengers experienced car sickness while they traveled and associated risk factors. Based on such assumptions, as mentioned earlier, this study presented passengers' car sickness and its indicators in Table 1.

### 4.3. Bivariate Analysis of Associated Factors and Car Sickness

The distribution of study participants who experienced car sickness and the corresponding *pvalues* of the chi-square test of association are presented in Table 2. As summarized in Table 2, the study participants who were from the age from 20 to 40 years experienced more car sickness with 87 (22.7%), followed by study participants from the age of 41 to 60 years with 36 (9.3%). Concerning sex, 82 (21.4%) of females experienced car sickness in the past six months, while the rest of 65 (16.9%) were males.

Car sickness is also affected by other factors. For instance, due to overcrowding of the road transport, 74 (19.2%) of the study participants experienced car sickness, while 56 (14.5%) of them experienced car sickness because of worries about the travels. Moreover, due to poor ventilation of vehicles , 69 (17.9%) of the study participants experienced car sickness; last, as a result of traveling with a full stomach, 84 (21.8%) of the study participants experienced car sickness.

As a result of troubled travels, occasionally, 28 (7.2%) of the study participants experienced car sickness. Moreover, due to traveling by vehicles with aggressive drivers, 152 (39.5%) of the study participants experienced car sickness. Furthermore, because of involvement in substance use like alcohol use before the journey, 134 (34.8%) of the study participants experienced car sickness. Last, due to not getting enough sleep before the journey, 117 (30.4%) of the study participants experienced car sickness. Not only due to sleep deprivation but also due to eating heavy types of meals before traveling, 86 (22.3%) of the study participants experienced car sickness.

As a result of traveling on curved roads, 77 (20.1%) of the study participants experienced car sickness. Moreover, traveling with other persons in overcrowded vehicles , 125 (32.5%) of the study participants experienced car sickness. Traveling from place to place by sitting at the back seat of vehicles , 45 (11.7%) of the study participants experienced car sickness. Furthermore, due to listening to a high volume of music from the vehicles, 32 (8.3%) of the study participants experienced car sickness. As a result of lack of focus on horizons and troubled travels with speed, 48 (12.5%) and 67 (17.4%) of the study participants, respectively, experienced car sickness. Last, because of frequent and sudden braking effects , 18 (4.6%) of the study participants experienced car sickness.

### 4.4. Goodness of Fit of the Model

The Hosmer and Lemeshow statistics (*χ*^2^ = 14.074, *p* = 0.08) and the likelihood ratio chi-square test (*χ*^2^ = 160.205, *p* ≤ 0.001) are used to measure how good the model ﬁts and indicated that the model fitted well. The ratio of values to the degree of freedom of the Pearson statistics was 1.092 and the deviance statistics was 0.941 and they were greater than 0.05, indicating that the ﬁtting was good.

## 5. Multivariate Analysis

All covariates that checked significantly at 25% in the bivariate analysis considered in the multivariable logistic regression analysis [27]. The result of the multivariable analysis is presented in Table 4.

As presented in Table 4, the odds of experiencing car sickness by older passengers were 0.972 (AOR = 0.972, 95% CI: 0.947, 0.999) times less likely than those at lower ages. In other words, as age increases, the probability of experiencing car sickness decreases. The odds of experiencing car sickness by male passengers were 0.357 (AOR = 0.357, 95% CI: 0.0.190, 0.673) times less likely compared to being female. That is to say, the odds of experiencing car sickness by female passengers were worse than those males; also, the odds of experiencing car sickness by female passengers were 2.8 (1/0.357) times more likely than those males. The likelihoods of passengers experiencing car sickness as a result of traveling by vehicles with aggressive drivers were 5.467 (AOR = 5.467, 95% CI: 2.456, 12.172) times higher than those traveling with friendly drivers.

Eating heavy meals before the journey by vehicles was also another variable, which predicted the occurrences of car sickness. The odds of experiencing car sickness by passengers who ate heavy meals before the journey were 4.147 (AOR = 4.147, 95% CI: 1.659, 10.366) times more likely than those who ate light meals. The sleeping status of the passengers had a significant effect on car sickness. The likelihoods of passengers experiencing car sickness due to deprived sleeping before the journey were 8.540 (AOR = 8.540, 95% CI: 2.575, 28.328) times more likely than those who slept sufficiently. Last, the likelihood of passengers experiencing car sickness as a result of traveling stickily by vehicles with other passengers were 9.521 (OR = 9.521, 95% CI: 5.194, 17.455) times more likely than those traveling freely.

## 6. Discussion

This study aimed to assess the associated risk factors inducing car sickness of passengers traveling by vehicles. How and why the signs and symptoms which indicated car sickness developed during traveling by vehicles were explained. The results indicated that the most types of signs and symptoms which manifested car sickness were nausea and headache. Moreover, vomiting and (cold) sweating were other signs and symptoms that manifested car sickness. Furthermore, the analyses were made based upon the results obtained from multivariate logistic regression. Last, this study discussed the relative effects of variables on car sickness of passengers traveled by vehicles in light of the related literature.

The results of this study indicated the passengers who were younger experienced more car sickness than those older. The results of this study were consistent with the findings of previous studies [1, 2, 8, 15]. Experiencing car sickness by children up to 2 years old was rare due to their body immunity system [15]. Likewise, people around the age of 50 years and above were uncommon to experience car sickness as a result of the biological effects [2, 8, 29]. Furthermore, car sickness occurred more frequently in people between the age of 2 and 12 years [2, 8]. All studies described the occurrence or not of car sickness in proportion to biological features rather than associated risk factors.

Because of older people experiencing more health problems than younger people, they were more likely to engage in prevention of risky health behaviors than younger people [30]. Moreover, they were much more likely to visit health care institutions for health services than younger adults. For such reasons, older people who traveled by a car might be less likely to have car sickness than those younger. In other words, older people traveling by car were more likely to practice healthy behaviors continuously and reduce less risky behaviors than younger people.

Concerning sex, there were differences between men and women in the sensation of car sickness. Gender is the sociocultural traits associated with being either male or female. The results of this study showed that women experienced more car sickness than men. The results are consistent with the previous existing literature [1, 14, 31]. Owing to the social roles, women had more illnesses or disease than men [31]. Such consequences or responses were not due to the biological effects. Moreover, the study indicated that in male-controlled (patriarchal) societies, women intentionally or unintentionally were forced to take predominantly the role of housewife or house workers. Because of such roles, they were busier than men by unpaid domestic work to support, care and feed the whole family. In such societies, unpaid domestic work is considered as a normal social role of women. As a result of such roles, women might be not getting enough physical rest or sleep very well compared to men before the journey. Currently, Ethiopia can be characterized asone of the patriarchal societies. Such types of social roles, circumstances, and conditions of women defined the country very well. Consequently, because of not taking enough sleep or rest before trips, women might develop headaches and cold sweating while traveling. Thus, as a result of such consequences or responses, generally women experienced more car sickness than men.

The other variable that determined the occurrences of car sickness was the personal behavior of the drivers of vehicles. The results indicated that the odds of experiencing car sickness were more when passengers traveled by vehicles with aggressive drivers. Aggressive drivers are individuals who are driving vehicles in unexpected ways or inappropriate manners. Consequently, these drivers drove more speedily than passengers ever faced before, and they braked more often and in inappropriate ways. Thus, such kinds of situations or events that occurred due to aggressive behaviors could result in worrying about the journey and developing anxiety and headache during the journey. Furthermore, as a result of such behavior, the drivers might be noticed by passengers as they were with less experience of driving. In addition, it was clear and witnessed that if passengers traveled in a car with such a type of a driver, the likelihood of being in stress and fear was expected [6, 32]. Stress is an emotional and mental tension, which results from very adverse circumstances or situations. Nevertheless, friendliness drivers are individuals who have good behavior, sociability, and experience much more about driving vehicles on road transport [6, 24, 28]. Thus, passengers who traveled in vehicles with such types of drivers had experienced less probability of sensing car sickness.

Concerning personal behavior, there were certain types of customs or traditions, which were practiced before journeys that ended up with car sickness. For instance, the type of meals which passengers had before their journey was taken as one of the traditions or customs. The results indicated that having heavy meals before travel could result in experiencing car sickness than having light meals. The results are consistent with the existing literature [8, 33–36]. Individuals have different traditions or customs due to socialization in general and understanding or internalization in particular. Explicitly, human beings have their own beliefs and practices on the preferences of foods or meals before or while they traveled [33]. Heavy meals are foodstuffs that are eaten in a large quantity and not digested easily within a short time [33]. In the study area, most types of heavy meals eaten by passengers were roasted beef meat, fried eggs, and dairy products.

It was suggested that if passengers avoid having heavy meals before traveling, the sensation of car sickness to some extent is reduced [33]. Thus, as a result of such qualities and practices of heavy meals, passengers could experiencethe sensation of car sickness. Not only heavy meals but also using alcohol for digestion purposes exacerbated the sensation of car sickness. Last, passengers who involved in such practices before traveling experienced car sickness due to the disturbances of the gastrointestinal tract [8, 35]. In effect, due to such personal behavior, passengers experienced nausea and vomiting during travel by vehicles.

Sleep deprivation was another important variable which contributed to experiencing car sickness. The results were consistent with the existing literature [33, 37–40]. Owing to not getting a natural state of rest in which eyes closed and body was inactive, passengers had a headache and (cold) sweating while they traveled by vehicles. Moreover, passengers who experienced car sickness during their trips had a problem with disturbances of sleeping after the journey for 24 hours [2, 6]. Sleep deprivation might be the result from early waking up for journeys, later going to bed, and not taking enough physical rest before starting traveling. Thus, as a result of such casualties, passengers traveled by vehicles had experienced car sickness.

The results indicated the type of journeys in line with how passengers traveled by vehicles determined the outcomes of car sickness. Passengers experienced car sickness when traveling with other persons in overcrowded vehicles. Traveling with other passengers in overcrowded situations means making journeys by vehicles that carry passengers beyond their carrying capacity. The results were consistent with the existing literature [8, 34, 37]. When passengers traveled in such conditions, the probability of facing discomforts and getting rid of foul air was high. It was obvious that in such conditions, the passengers traveled with poor ventilation that made less comfort, and got rid of foul air. Furthermore, as a result of the windows of certain vehicles not functioning and some passengers not interested in opening the window, the exposure to fresh air for purifying was less. In such situations, passengers traveled certain distances without safe travels, which made them face unpleasant events [4, 40, 41]. While they traveled in such kinds of situations, they developed headaches and (cold) sweating that indicated car sickness.

## 7. Limitations and Strengths of the Study

This was the study that aimed to investigate the association between sociocultural, situational, and individual behavioral factors and passengers' car sickness experienced while traveling by vehicles. However, the study was not without some limitations. One of the limitations was about cautiousness that came with any survey employing a sample of convenience with self-reported behaviors that could not verify or measure accurately . However, it was considered to be a valid method of investigating sociocultural, situational, and individual behavioral factors for car sickness. Another limitation of the study was the potential sampling bias due to the use of convenience sampling, which might not be representative of the entire passengers in the study area. However, an attempt was made to reduce the sampling bias by distributing a survey to a large number of passengers waiting in the bus stations with the consideration of their sex, and age, as well as distributing a survey proportionally to all clusters of the study area for a long period of time. An investigation into the association between associated risk factors and car sickness of passengers traveled by vehicles that might be used by policymakers and other concerned bodies or professionals for the countermeasures was taken into account as the strength of the study.

## 8. Conclusions

The results indicated that car sickness was affected by associated risk factors which emanated from sociocultural influences, situational circumstances, and individual behaviors. As a result of being less likely to practice healthy behaviors continuously and less likely to reduce risky behaviors, younger passengers were affected more by car sickness compared with older ones. Typically, women as a result of being busy with domestic unpaid work they had not taken enough rest, and not slept well before traveling. The other reason for passengers experiencing more car sickness was sleep deprivation due to early waking up for journeys, later going to bed, and not taking enough physical rest before travels. Eating heavy types of meals such as roasted beef meat, fried eggs, and dairy products triggered the sensation of car sickness. Furthermore, passengers traveling with other passengers in overcrowded vehicles from place to place could experience more car sickness. Last, the passengers who traveled by vehicles with aggressive drivers could experience more car sickness. 

## 9. Recommendations

In light of the above findings, the following pertinent recommendations were forwarded. This study suggested that young passengers should take medications that reduce the sensation of car sickness, and women should reduce unpaid domestic work, take enough rest, and sleep well before they start traveling. Also, this study suggested passengers during their travel should request aggressive drivers to drive slowly and uniformly or consistently. Furthermore, this study pointed out that passengers should avoid eating heavy meals during travels. Finally, not only women but also other passengers should take good enough physical rest and sleep well before traveling. The authors recommended future studies to incorporate more variables for further investigations than the current ones; additional studies to be needed with more comprehensive investigations by considering the carrying capacity of vehicles and the permitted distances that vehicles moved for identifying the associated risk factors that could result more in countermeasure to car sickness. Lastly, the authors strongly recommended further studies that address explicitly a type of vehicle, either as a small or medium or large, to be used by passengers. 

## Figures and Tables

**Table 1:** Distribution and indicators of car sickness.

Variables	Categories	*n*	%
Passengers experienced car sickness	Yes	147	38.3
	No	237	61.7
Indicators of car sickness of passengers	Vomiting	36	9.4
	Cold sweating	27	7.03
	Headache	41	10.7
	Nausea	**43**	**11.2**

Source: Authors Survey, 2019.

**Table 2:** Bivariate analysis of associated factors and car sickness.

Variables	Experienced car sickness			*p* value
	Categories	*n*	%	
Age (in years)	<20	16	4.2	<0.001
	20–40	87	22.7	
	41–60	36	9.3	
	>60	8	2.1	
Sex	Female	82	21.4	<0.001
	Male	65	16.9	
Overcrowding of roads	Yes	74	19.2	<0.001
Worrying about travels	Yes	56	14.5	0.013
Poor ventilation of vehicles	Yes	69	17.9	0.023
Full stomach	Yes	84	21.8	0.014
Occasionally travels	Yes	28	7.2	0.020
Aggressive drivers	Yes	152	39.5	<0.001
Substance use	Yes	134	34.8	<0.001
Sleep deprivation	Yes	117	30.4	0.010
Heavy type of meals	Yes	86	22.3	0.017
Curved roads	Yes	77	20.1	0.013
Overcrowded journeys	Yes	125	32.5	0.004
Sitting at the back seat of vehicles	Yes	45	11.7	0.006
Listening a high volume music	Yes	32	8.3	0.021
Lack of focus on horizon	Yes	48	12.5	0.018
Speedily traveled by vehicles	Yes	67	17.4	0.012
Braking effects of vehicles	**Yes**	**18**	**4.6**	**0.024**

Source: Authors Survey, 2019.

**Table 3:** Sociodemographic characteristics of passengers.

Variables	Categories	*n*	%
Sex	Female	129	33.6
	Male	255	66.4
Age (in year)	<20	42	10.9
	20–40	172	44.8
	41–60	98	25.5
	>60	72	18.8
Occupation	Merchant	103	26.8
	Employee	135	35.2
	Unemployed	89	23.2
	Students	57	14.8

Source: Authors Survey, 2019.

**Table 4:** Logistic regression predicting car sickness of passengers.

Covariates/Variables	Categories	*β*	Sig.	AOR	95% CI for AOR	
					Lower	Upper
Age	Year	−.0028	0.042^*∗*^	0.972	0.947	0.999
Sex	Male	−1.029	0.001^*∗*^	0.357	0.190	0.673
	Female	0^a^	.	1	.	.
Reasons	Overcrowding of roads	−0.747	0.138	0.474	0.176	1.271
	Worrying about journeys	−0.624	0.257	0.536	0.182	1.577
	Poor ventilation of vehicles	−0.176	0.746	0.838	0.289	2.435
	Normal circumstances	0^a^	.	1	.	.
Stomach status of individuals	Full	−0.627	0.092	0.534	0.257	1.109
	Empty	0^a^	.	1	.	.
Occasionally travels	Yes	0.064	0.826	1.066	0.605	1.879
	No	0^a^	.	1	.	.
Personal behaviors of drivers	Aggressiveness	1.699	0.000^*∗*^	5.467	2.456	12.172
	Friendliness	0^a^	.	1	.	.
Type of meals	Heavy meals	1.422	0.002^*∗*^	4.147	1.659	10.366
	Light meals	0^a^	.	1	.	.
Sleeping status before journey	Deprived	2.145	0.000^*∗*^	8.540	2.575	28.328
	Sufficient	0^a^	.	1	.	.
Substance use	Yes	1.123	0.090	3.073	0.838	11.267
	No	0^a^	.	1	.	.
Nature of roads	Curved roads	−0.347	0.264	0.707	0.384	1.300
	Straight roads	0^a^	.	1	.	.
Nature of journeys	Overcrowded	2.254	0.000^∗^	9.521	5.194	17.455
	Freely	0a	.	1	.	.
Sitting at the back seat of vehicles	Yes	−0.163	0.715	0.850	0.354	2.039
	No	0^a^	.	1	.	.
Listening high volume of music	Yes	−.069	0.911	.934	0.279	3.128
	No	0^a^	.	1	.	.
Lack of focused at horizon	Yes	0.578	0.065	1.783	0.964	3.298
	No	0^a^	.	1	.	.
Speed of the vehicles	Yes	−0.316	0.327	0.729	0.388	1.371
	No	0^a^	.	1	.	.
Braking effects of vehicles	Yes	0.148	0.652	1.159	0.609	2.206
	**No**	**0 ** ^**a**^	.	**1**	.	.

(LRT = 160.205, *p*-value < 0.001), (HLT = 14.074, *p*-value = 0.08), ^*∗*^ significant at *p* ≤ 0.05.

## Data Availability

The data sets used and/or analyzed during the current study are available from the corresponding author on a reasonable request.
